# Physical inactivity, gender and culture in Arab countries: a systematic assessment of the literature

**DOI:** 10.1186/s12889-018-5472-z

**Published:** 2018-05-18

**Authors:** Eman Sharara, Chaza Akik, Hala Ghattas, Carla Makhlouf Obermeyer

**Affiliations:** 1 Center for Research on Population and Health, Faculty of Health Sciences, American University of Beirut, P.O. Box 11-0236/EPHD, Riad El Solh, Beirut, Lebanon; 2 Institute for Advanced Study, Aix-Marseille University, Marseille, France

**Keywords:** Physical activity, Social determinants, Gender, Culture, Arab countries

## Abstract

**Background:**

Physical inactivity is associated with excess weight and adverse health outcomes. We synthesize the evidence on physical inactivity and its social determinants in Arab countries, with special attention to gender and cultural context.

**Methods:**

We searched MEDLINE, Popline, and SSCI for articles published between 2000 and 2016, assessing the prevalence of physical inactivity and its social determinants. We also included national survey reports on physical activity, and searched for analyses of the social context of physical activity.

**Results:**

We found 172 articles meeting inclusion criteria. Standardized data are available from surveys by the World Health Organization for almost all countries, but journal articles show great variability in definitions, measurements and methodology. Prevalence of inactivity among adults and children/adolescents is high across countries, and is higher among women. Some determinants of physical inactivity in the region (age, gender, low education) are shared with other regions, but specific aspects of the cultural context of the region seem particularly discouraging of physical activity. We draw on social science studies to gain insights into why this is so.

**Conclusions:**

Physical inactivity among Arab adults and children/adolescents is high. Studies using harmonized approaches, rigorous analytic techniques and a deeper examination of context are needed to design appropriate interventions.

**Electronic supplementary material:**

The online version of this article (10.1186/s12889-018-5472-z) contains supplementary material, which is available to authorized users.

## Background

Global increases in body mass index, raised blood pressure and cardiovascular disease have been attributed in part to the reduction in physical activity resulting from changes in the organization of labor and transportation, and to increases in sedentary behavior. The evidence on the magnitude of these changes and their consequences for health is well recognized. The World Health Organization (WHO) ranks physical inactivity as the fourth leading cause of global mortality, estimating that it results in 3.2 million deaths globally, mainly due to cardiovascular disease, diabetes, hypertension, and some cancers [1–6]. Analyses of the Global Burden of Disease estimate that insufficient physical activity accounts for an estimated 13.4 million disability adjusted life years (DALYs) related to ischemic heart disease, diabetes and stroke [7].

There are major variations in the prevalence of physical inactivity across regions and among countries. In the Arab region, alarming predictions have been made in light of very unfavorable combinations of risk factors related to body mass index, its determinants including physical activity, and its health consequences [8–10]. Some studies have compared indicators across countries [11–15], but there have not been comprehensive assessments of the prevalence and determinants of physical inactivity across the Arab region. Yet, such regionally specific assessments are key to identify patterns and formulate interventions, and would be especially timely, given mounting evidence on the health effects of sedentary behaviour and physical inactivity, the growing awareness of the need for population interventions, and the urgency of scaling up policies and programs to increase physical activity in low and middle income countries [16]. In addition, there is a need to go beyond simplistic explanations of observed patterns in terms of religion or education.

Hence, this study was designed to review research on the subject, assess levels and variability in physical inactivity across countries and social groups, and gain insights into the extent to which social determinants, in particular those related to gender, could explain such unfavorable indicators. The diversity of indicators and measures in the region, and the difficulty of obtaining original survey data precluded the possibility of conducting a systematic review or meta-analysis. But we thought it was important to take stock of what was known about physical inactivity in the region and to review the explanations that are offered for observed levels, in order to identify patterns and to inform policies designed to increase physical activity.

The review proceeds as follows. We first present a summary of the evidence from studies published in peer-reviewed journals, including the availability and comparability of studies and the instruments used. Secondly, we provide a synthesis of prevalence levels based on the reports of surveys that have used standardized definitions and measurements. We then bring together the results of studies that examined the social determinants of physical activity, with special attention to those related to gender and cultural factors. Lastly we draw the implications of these results for research and policies.

## Methods

### Search strategy and inclusion criteria

We sought to retrieve research published in refereed journals and reports of surveys, and our approach was three-pronged. First, we searched for articles in refereed journals investigating physical inactivity in countries of the Arab region, published between January 2000 and January 2016, in MEDLINE, Popline and Social Sciences Citation Index (SSCI) databases. Various combinations of MeSH terms and key words were used, related to physical activity/ inactivity, sedentary lifestyle, exercise, sports, its prevalence, incidence, epidemiology, the burden it represents, and social or cultural factors. Details are shown in Additional file 1. Studies published in any language were retrieved. Two researchers conducted title and abstract screening, followed by full-text screening, checking to harmonize results regarding inclusion or exclusion; disagreements were discussed by the team as a whole and resolved. This was done according to the Assessing the Methodological Quality of Systematic Reviews (AMSTAR) appraisal tool for systematic reviews [17]. In addition to the electronic search, we searched reference lists of the articles identified.

Sources were included if they fulfilled the following criteria: assessed physical activity or inactivity as an outcome or a determinant; were conducted among residents of Arab countries (the 22 countries of the Arab League: Algeria, Bahrain, Comoros, Djibouti, Egypt, Iraq, Jordan, Kingdom of Saudi Arabia (KSA), Kuwait, Lebanon, Libya, Mauritania, Morocco, Oman, Palestine, Qatar, Somalia, Sudan, Syria, Tunisia, United Arab Emirates (UAE), and Yemen); described the design and methods; reported on sample size; described how physical activity/inactivity was measured; reported on the prevalence of physical activity/inactivity. Multi-country studies were included if they presented data on at least one Arab country. Studies conducted exclusively on patients with a particular disease diagnosis, and studies conducted on Arabs residing outside the Arab region were excluded. To be included, articles needed to fulfill quality criteria informed by the Preferred Reporting Items for Systematic Reviews and Meta-Analyses (PRISMA) guidelines [18], including clear eligibility criteria for study selection, description of information sources, data and variables; we excluded studies that did not report on sample size, age range of study population, and those that presented unclear or inconsistent numbers.

Secondly, we retrieved the reports of surveys on physical activity conducted by international organizations in collaboration with country partners; these surveys generally use standardized instruments and the two main sources are the World Health Organization (WHO) surveys on non-communicable disease risk factors (STEPS) which include modules on physical activity among adults; and the Global School-based Student Health Surveys (GSHS) which measure activity among adolescents. We present results separately for studies based on national surveys using standardized definitions and measures, and whose results represent comparable and higher-quality estimates.

A third part of the review was to retrieve data from sources that considered physical inactivity in relation to social factors such as age, marriage, education, employment, residence, and those that examined cultural and social barriers to physical activity. We sought to gain insights into the socio-cultural context of physical activity, and to explain the patterns that emerged from the analysis of the quantifiable data. We extracted notes and themes from those sources that included qualitative information, and provide a critical synthesis of main findings. Thus, this review draws both on rigorous quantitative analyses and a narrative synthesis of qualitative studies.

### Data extraction and analysis

Citations from search results of databases were imported into the reference manager EndNote and duplicates removed. We used the open-source Open Data Kit (ODK) (https://ona.io/) to create the data entry protocol. The data extracted for each study included: (1) article identification (title, author/s, publication year, journal, country/ies of study); (2) research design, setting, sample size, study population, gender, and age; (3) definition of physical activity/inactivity, instrument used, reported prevalence; and, (4) demographic, economic, lifestyle and social correlates of physical inactivity. In addition, we retrieved themes from those studies that examined the social context of physical activity and provided information about gender and cultural differences.

We retrieved the most recent data from STEPS and GSHS surveys. For countries where no published reports were available, we retrieved any data available from the WHO website.

Regarding the outcome variable, because of the diversity of definitions and measures of physical activity, we found that the most consistent way to report the results was to use physical inactivity, which refers to not engaging in any physical activity and/or being in the lowest category of physical activity, however physical activity was defined in the study. This is consistent with other studies that have reviewed physical activity across the world [13].

We present results separately for adults and for children/adolescents. We defined as adults those respondents aged 18 or older, or those who were categorized as adults in the articles; younger respondents were categorized as children/adolescents. In the discussion, we build on the narrative synthesis of qualitative studies.

## Results

### The evidence on physical inactivity

#### Sources and quality of data

**Fig. 1:**
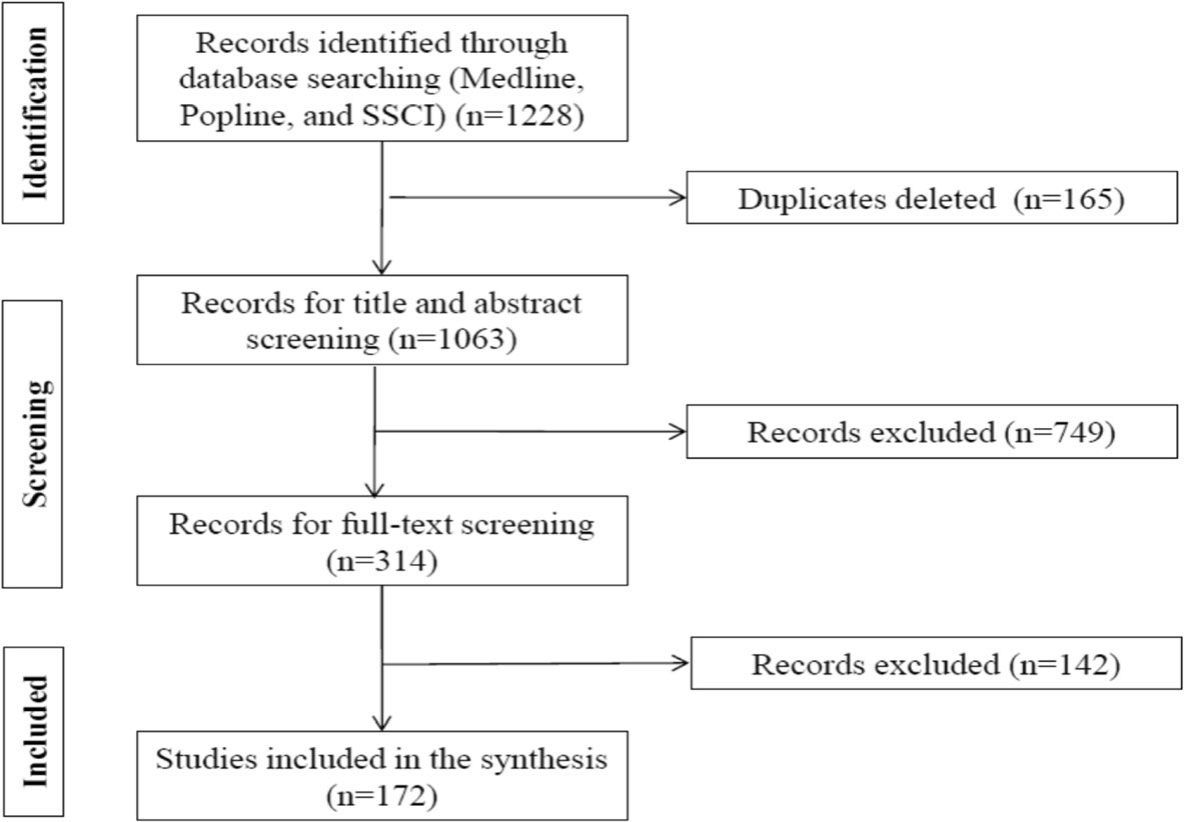
Flow chart of the review’s inclusion and exclusion process

Our search retrieved 1,228 articles, of which 172 met the inclusion criteria. Figure 1 provides a flow chart of the review’s inclusion and exclusion process. The included articles referred to a total of 157 datasets: 149 from studies conducted in a single country and 8 conducted as part of multi-country studies; the results of multi-country studies are counted once for each individual country. Some articles were based on the same datasets, including six articles based on STEPS and GSHS surveys. Only 16/143 journal articles reported on surveys using nationally representative samples; qualitative data were retrieved from five qualitative studies and from four mixed methods studies.

All STEPS and GSHS, and 125/157 journal articles include both men/boys and women/girls. GSHS surveys (usually on adolescents 13-15) have been conducted in all but four countries of the region (Bahrain, Comoros, KSA, and Somalia). STEPS surveys usually include adults aged 25-64. Age categories in journal articles are more diverse. 12 countries had both STEPS and GSHS surveys. Unlike GSHS, not all STEPS were based on nationally representative samples (exceptions were Algeria, Mauritania, Oman and Sudan). Additional results about the prevalence of physical inactivity and its determinants are available from journal articles that used the World Health Surveys (WHS) as data sources. STEPS are based on household surveys and GSHS on school populations, while the settings in journal articles included schools (28%), health facilities (27%), households (16%), and universities (15%).

Table 1 shows disparities in the available evidence: for some countries there are very few studies (Algeria, Comoros, Djibouti, Iraq, Somalia, Sudan and Yemen), while for others many more sources are available (for example 40 for Saudi Arabia). There is also a variability in sample size, with most studies in the range of 200-2000 and a few large studies including several thousand respondents.

**Table 1:** The evidence on physical activity in Arab Countries: studies, sample sizes and instruments

Country	Total number of studies	Data from Reports/Factsheets^a^		Data from Journal Articles				
		Studies (#)	Sample size/range	Single country studies (#)	Multi-country studies (#)	Sample size/range	Nationally representative studies (#)	Instruments used^b,c^
Algeria	4	2 [116, 117]	4102 – 4532	1 [118]	1 [15]	293 – 4698^d^	-	Locally Validated Questionnaire [15]
Bahrain	4	1 [119]	1769	4 [82, 120–122]	0	142 – 2013	1 [122]	WHO Heart and Health Questionnaire [120]
Comoros	3	1 [123]	5556	0	2 [12, 13]	1492 – 212021^d^	-	IPAQ [12, 13]
Djibouti	1^e^	1 [124]	1777	0	1 [11, 58]	829 – 882	1 [58]	PACE+ [11, 58]
Egypt	12^e^	2 [125, 126]	2568 – 5300	7 [34, 46, 74, 127–130]	4 [11, 21, 58, 131, 132]	188 – 3271	1 [58]	IPAQ [131] PACE+ [11, 58]
Iraq	3	2 [133, 134]	2038 – 4120	1 [135]	0	200	-	-
Jordan	15^e^	2 [136, 137]	2197 – 3654	12 [61, 64, 70, 72, 80, 81, 138–143]	2 [11, 15, 58]	209 – 8791	3 [58, 138, 141]	PACE+ [11, 58] ATLS [140] Locally Validated Questionnaire [15]
KSA	48	1 [144]	3547	46 [19, 20, 24–27, 36, 38, 41, 42, 44, 48, 51–54, 65, 66, 68, 69, 73, 93, 96, 98, 99, 103, 107, 145–167]	1 [12]	30 – 197681	3 [38, 157, 167]	ATLS [24, 42, 66, 98, 99, 167] Barriers to Being Active Quiz: CDC website [44] CDC Adolescent Health Survey [27] Electronic Pedometer [19, 20] GPAQ [36, 54, 107, 150] IPAQ [12, 26, 69, 93, 157] KPAS [25] WHO stepwise questionnaire [166] YRBSS and GSHS Questionnaires [167]
Kuwait	15	2 [94, 168]	2280 – 3637	12 [37, 49, 79, 97, 169–177]	1 [15]	224 – 38611	3 [169–171]	The Exercise Pattern Questionnaire [172] Locally Validated Questionnaire [15]
Lebanon	13^e^	2 [178, 179]	1982 – 2286	13 [22, 29, 30, 35, 57, 59, 63, 114, 180–186]	0	83 – 2608	5 [35, 181, 182, 185, 186]	IPAQ: 2 used a shorter version [35, 181, 182] Self-reported Weekly Activity Checklist [59]
Libya	5^e^	2 [187, 188]	2242 – 3590	2 [56, 189]	2 [11, 15, 58]	383 – 1300	1 [58]	Locally Validated Questionnaire [15]
Mauritania	4	2 [190, 191]	2063 – 2600	0	2 [12, 13]	2726 – 212021^f^	-	IPAQ [12, 13]
Morocco	6^e^	1 [192]	2924	5 [38, 61, 62, 75, 84, 85, 105, 193]	1 [11, 58]	239 – 2891	1 [58]	IPAQ [39] PACE+ [11, 58]
Oman	7^e^	2 [94, 194]	1373 –3468	5 [33, 71, 195–197]	2 [11, 58, 198]	10 – 5409	2 [58, 195]	GPAQ [33] GSHS Questionnaire [197] IPAQ [196] LASA Physical Activity Questionnaire [197] PACE+ [11, 58] WHO Health Behavior in School Children [196]
Palestine	11	2 [126, 199, 200]	1908 – 6957	8 [55, 77, 86, 100, 201–204]	1 [15]	16 – 8885	1 [202]	MESA [204] Locally Validated Questionnaire [15]
Qatar	10^e^	2 [205, 206]	2021 – 2496	9 [23, 40, 207–214]	0	340 – 2467	1 [214]	GPAQ [214]
Somalia	1	0	-	1 [215]	0	173	-	-
Sudan	3	2 [216, 217]	1573 – 2211	1 [218]	0	1200	-	-
Syria	4	1 [219]	3102	2 [92, 220, 221]	1 [15]	1168-2037	-	Locally Validated Questionnaire [15]
Tunisia	12^e^	1 [222]	2870	9 [31, 32, 43, 76, 223–228]	4 [11–13, 58, 131]	10 – 17789	2 [43, 223]	IPAQ [12, 13, 228], (including 1 short version) PACE+ [11] Locally Validated questionnaire [31, 43, 223, 224]
UAE	15^e^	1 [229]	2581	11 [28, 45, 47, 67, 78, 83, 96, 230–233]	4 [11–13, 15, 58]	20 – 9918	1 [58]	Health Promoting Lifestyle Profile [47] IPAQ: 3 used shorter version [12, 13, 78, 83] PACE+ [11, 58] Locally Validated Questionnaire [15]
Yemen	1^e^	1 [234]	1175	0	1 [11]	568	1 [11]	PACE+ [11]

^a^WHO-STEPS and GSHS used GPAQ and PACE+ respectively to assess physical inactivity

^b^This column indicates whether some studies used internationally or locally standardized/validated instruments, with the reference number in brackets; where not indicated, the assessment of physical activity was either not specified or based on a single question

^c^ATLS: Arab Teens Lifestyle Study – GSHS: Global School-based Student Health survey – IPAQ: International Physical Activity Questionnaire – KPAS: Kaiser Physical Activity Survey –LASA: Longitudinal Aging Study Amsterdam – MESA: Multi-Ethnic Study of Atherosclerosis questionnaire – PACE+: Patient-Centered Assessment and Counseling for Exercise Plus Nutrition – YRBSS: The Youth Risk Behavior Surveillance System

^d^For multi-country studies where the information on sample size was not available for each country, we included the pooled sample size.

^e^A number of journal articles are based on WHO surveys (STEPS and GSHS)

STEPS and GSHS use standardized instruments, namely the Global Physical Activity Questionnaire (GPAQ) and the Patient-Centered Assessment and Counseling for Exercise Plus Nutrition (PACE+) respectively, but only 38/143 journal articles referred to studies that used validated instruments. About half of these used internationally validated tools, such as the International Physical Activity Questionnaire (IPAQ), the GPAQ or the PACE+; others used regionally or nationally validated questionnaires. Two studies used electronic pedometers [19, 20]. The majority of studies (112/157) simply used respondents’ reports. Only five studies followed the WHO’s recommendations regarding the multi-dimensional categorization of physical activity into work, active transportation, household and family, and leisure-time activities; the questionnaires that follow this recommendation include the long version of IPAQ, the GPAQ, and the Kaiser Physical Activity Survey (KPAS).

#### Prevalence of physical inactivity

Tables 2 and 3 present the prevalence of physical inactivity among adults; Table 2 summarizes data from WHO-STEPS surveys and Table 3 presents results of journal articles. Among adults, the prevalence of physical inactivity defined as performing less than 600 MET-minute per week, exceeded 40% in all Arab countries except for Comoros (21%), Egypt (32%) Jordan (5%); it reached 68% in KSA (national) and 87% in Sudan (subnational).

**Table 2:** Prevalence of physical inactivity among adults based on data from WHO-STEPS surveys

Country^a^	Year of study	Age range	Sample size	Prevalence of Physical inactivity
National samples				
Comoros	2011	25-64	5556	20.1
Egypt	2011-2012	15-64	5300	32.1
Jordan	2007	18+	3654	5.2
Iraq	2015	18+	4120	47.0
Kuwait	2014	18-69	4391	62.6
Libya	2009	25-64	3590	43.9
Lebanon	2008	25-64	1982	45.8
Palestine	2010-2011	15-64	6957	46.5
Qatar	2012	18-64	2496	45.9
Saudi Arabia	2005	25-64	3547	67.6
Subnational samples				
Algeria	2003	25-64	4102	40.7
Mauritania	2006	25-64	1971^b^	51.3
Sudan	2005-2006	25-64	1573	86.8

^a^For Bahrain and Oman, surveys were available but no total physical inactivity prevalence could be retrieved; specific prevalence of work, transportation, and leisure time were 71.9%, 63.9%, and 57.1%., respectively for Bahrain and 6.4%, 30.1%, and 53.8% for Oman

^b^Sample size was calculated for age group (25-64) from numbers provided in the report

**Table 3:** Prevalence of physical inactivity among adults based on findings from published literature

Country	First author, year (year of study)	Source	Definition	Instrument	Prevalence (%)	Age range	Sample size
National samples							
Comoros	Guthold, 2008 (2002-2003)	World Health Survey	<600 MET-minutes/week	IPAQ	2.7	18-69	1492
Jordan	Zindah, 2008 (2004)	Behavioral Risk Factor Surveillance System	Not engaging in moderate activity (resulting in light sweating, small increases in breathing or heart rate.	NA	51.8	18+	710
Kuwait	Ahmed, 2013 (2002-2009)	National Nutrition Surveillance Data	No deliberate non-work related exercise outside the home such as walking, running or cycling	NA	68.4	20+	32811
	Al-Zenki, 2012 (2008-2009)	NA	Neither moderately nor very active^a^	NA	77.1	20+	765
	Alarouj, 2013 (NA)	NA	Neither moderate nor vigorous physical activity^a^	NA	63.0	20-65	1970
KSA	Al-Baghli, 2008 (2004-2005)	NA	No physical activity or mild physical activity (ordinary housework, walking)	NA	79.2	30+	197681
	Al-Nozha, 2007 (1995-2000)	Coronary Artery Disease in Saudis Study (CADISS)	<600 MET-minutes/week	NA	96.1	30-70	17395
	Memish, 2014 (2013)	Saudi Health Information Survey	Neither moderate nor vigorous physical activity^a^	IPAQ	69.1	15+	10735
Lebanon	Farah, 2015 (2013-2014)	NA	Neither moderate-intensity physical activity for at least 150 min per week or vigorous intensity physical activity for 75 min at least per week	NA	76.0	40+	1515
	Tohme, 2005 (2003-2004)	NA	Less than 30 min of physical exercise	NA	40.3	30+	954
Mauritania	Guthold, 2008 (2002-2003)	World Health Survey	<600 MET-minutes/week	IPAQ	61.9	18-49	1492
Morocco	El Rhazi, 2011 (2008)	NA	Less than 30 min per day		38.7	18+	2620
	Najdi, 2011 (2008)	NA	<3METs	IPAQ	16.5	18-99	2613
Palestine	Baron-Epel, 2005 (2002-2003)	KAP and EUROCHIS^&^	Exercising less than once per week for at least 20 consecutive minutes^b^	NA	62.8	21+	1826^c^
Tunisia	Guthold, 2008 (2002-2003)	World Health Survey	<600 MET-minutes/week	IPAQ	14.6	18-69	4332
UAE	Guthold, 2008 (2002-2003)	World Health Survey	<600 MET-minutes/week	IPAQ	43.2	18-69	1104
Subnational samples^d^							
Bahrain	Al-Mahroos, 2001 (NA)	NA	<1 km walking	WHO Heart and Health Questionnaire	77.5	40-69	2013
	Hamadeh, 2000 (NA)	NA	No exercise	NA	89.1	30-79	516
Egypt	Abolfotouh, 2007 (2002-2003)	NA	No non-vigorous physical activity for at least 20 minutes or 3 times per week	NA	33.8	17-25	600
	Kamel, 2013 (2010-2011)	NA	NA	NA	63.8	60+	340
	Mahfouz, 2014 (2011)	NA	No exercise	NA	78.3	NA	300
Jordan	Centers for Disease, Control, Prevention, 2003 (2002)	Jordan Behavioral Risk Factor Survey	Less than having moderate: activity that caused light sweating and small increases in heart rate or breathing for 30 minutes	NA	47.4	18+	8791
	Mohannad, 2008 (2002)	NA	No activity that caused light sweating and small increases in heart rate or breathing	NA	58.7	40+	3083
	Kulwicki, 2001 (NA)	NA	No exercise	NA	22.5	17-93	209
	Madanat, 2006 (2003)	NA	<30 mins of physical activity/week	NA	81.5	Mean: 21.1	431
KSA	Almurshed, 2009 (2003-2004)	NA	No exercise	NA	52.0	30+	50
	Al-Quaiz, 2009 (2007)	NA	Not practicing in any regular sport and leisure time physical activity	CDC web site questionnaire	82.4	15-80	450
	Al-Senany, 2015 (NA)	NA	Less than one hour weekly activity	NA	69.0	60-90	55
	Amin, 2011 (NA)	NA	<600 MET-minutes/week	GPAQ	48.0	18-64	2176
	Amin, 2014 (NA)	NA	<30 minutes /≥ 5 days/week	GPAQ^e^	80.0	18-78	2127
	Awadalla, 2004 (2012-2013)	NA	Neither vigorous: >6 METs nor moderate: 3-6 METs	IPAQ (short form)	58.0	17-25	1257
	Garawi, 2015 (2004-2005)	NA	<600 MET-minutes/week	GPAQ	67.0	15-64	4758
Kuwait	Naser Al-Isa, 2011 (NA)^f^	NA	Not engaging in regular physical activity	NA	45.0	NA	787
Lebanon	Al-Tannir, 2008 (2007)	NA	Less than 3 days/week	NA	44.5	18+	346
	Musharrafieh, 2008 (2001)	NA	Physical exercise for <0.5 h/week	NA	73.6	Mean: 21.0	2013
	Tamim, 2003 (2000-2001)	NA	<3 hours/week	NA	64.3	Mean: 21.0	1964
Mauritania	Guthold, 2008 (2002-2003)	World Health Survey	<600 MET-minutes/week	IPAQ	61.9	18-49	2726
Palestine	Abdul-Rahim, 2003 (NA)	NA	Occupation-related sedentary-light PA for men AND no exercise for women	NA	56.2	30-65	936
	Abu-Mourad, 2008 (2005)	NA	No home exercise or sports	NA	78.0	18+	956
Qatar	Al-Nakeeb, 2015 (NA)	NA	<840 MET-min/week	NA	50. 8^g^	Mean= 21.2	732
	Bener, 2004 (2003)	NA	Not walking, cycling at least 30 minutes/day	NA	55.3	25-65	1208
Somalia	Ali, 2015 (2013)	NA	<2 hours/week	NA	33.5	18-29	173
Syria	Al Ali, 2011 (2006)	2nd Aleppo Household Survey	Less than 15 mins/ week of sport or brisk walking	NA	82.3	25+	1168
Tunisia	Maatoug, 2009 (2009)	NA	<150 mins/week of moderate level of physical activity	Oxford Health Alliance Community Intervention for Health Project	44.4	Mean: 37.9	1880
UAE	Abdulle, 2006 (2001-2005)	NA	Less than one hour, <3 times per week	NA	39.4	20-75	424^h^
	McIlvenny, 2000 (NA)	NA	No regular exercise	NA	54.0	18-94	254
	Sabri, 2004 (2001-2002)	NA	< 1 hour/week) of sport	NA	47.5	20-65	436

^a^Definition of physical activity not specified

^b^It includes: walking, running, swimming playing ball games or any other sports activities (combined every day and nearly every day with once or twice a week)

^c^Prevalence rate for Arabs only

^d^One study conducted in Libya by Salam (2012) was excluded from the prevalence table; it includes adolescents and youth (17-24 years) and the prevalence was 65.0%

^e^Combined Global Physical Activity Questionnaire (GPAQ) version 2.0 with a modified show card based on World Health Organization STEPs survey

^f^Kuwaiti college students

^g^Only Qatari students

^h^Only normotensives

Among the 102 journal articles on adults, 48 reported on prevalence among both men and women. In most countries, inactivity exceeded 40%; a few studies found lower inactivity, including nationally representative studies in Comoros (3%), Morocco (17%), and Tunisia (15%), and subnational studies in Egypt and Somalia (34%) and Jordan (23%).

Physical inactivity among children/adolescents is presented in Tables 4 and 5, based on GSHS reports (Table 4) and journal articles (Table 5). Prevalence of physical inactivity, defined in GSHS as <60 minutes per day on 5 or more days during the past seven days, is very high, with a low of 65% in Lebanon and a high of 91% in Egypt. Journal articles report similarly high levels of inactivity (>60%) except in KSA (45%) and Tunisia (29%), with smaller studies showing a wide variation within and among countries.

**Table 4:** Prevalence of physical inactivity among children/adolescents using data from Global School-based Student Health Surveys (GSHS)^a^

Country	Year of study	Age range	Sample size	Total prevalence of physical inactivity
Definition: < 60 mins per day on five or more days during the past seven days				
Iraq	2012	13-15	2038	80.0
Lebanon	2011	13-15	2286	65.4
Mauritania	2010	13-15	2063	83.7
Morocco	2010	13-15	2924	82.6
Palestine (Gaza Strip)	2010	13-15	2677	75.8
Palestine (West Bank)	2010	13-15	1908	81.7
Qatar	2011	13-15	2021	85.0
Sudan	2012	13-15	2211	89.0
Syria	2010	13-15	3102	84.9
UAE	2010	13-15	2581	72.5
Definition: < 60 mins per day on all 7 days during the past 7 days				
Djibouti	2007	13-15	1777	85.1
Egypt	2006	13-15	5249	90.6
Jordan	2007	13-15	2197	85.6
Kuwait	2015	13-17	3637	84.4
Libya	2007	13-15	2242	83.9
Oman	2015	13-17	3468	88.3
Tunisia	2008	13-15	2870	81.5
Yemen	2008	13-15	1175	84.8

^a^All based on nationally representative samples

**Table 5:** Prevalence of physical inactivity among children/adolescents using data from journal articles

Country	First author, year	Source	Definition	Questionnaire used	Prevalence (%)	Age range	Sample size
National samples							
Bahrain	Musaiger, 2014 (2006–2007)	NA	<5days/week of playing sport	NA	72.1	15-18	735
Egypt	Salazar-Martinez, 2006 (1997)	NA	Not engaged in sports	NA	62.3	11-19	1502
KSA	AlBuhairan, 2015 (NA)	NA	Complete absence of exercise	YRBSS and the GSHS Questionnaires^a^	45.2	Mean: 15.8	12575
Oman	Afifi, 2006 (2004)	NA	Engaging in physical activities <once per week, apart from school physical education	27-item Child Depression Inventory	66.3	14-20	5409
Palestine	Al Sabbah, 2007 (2003–2004)	Health Behavior in School-aged Children Survey	< 60 minutes/day, <5/7 days per week	WHO international HBSC questionnaire	80.0	12-18	8885
Tunisia	Nouira, 2014 (2009-2010)	NA	NA	Oxford Health Alliance for community intervention for health	88.1	12-14	3987
	Aounallah-Skhiri, 2012 2005	NA	< 3 Mets	Locally validated questionnaire	29.4	15-19	2870
Subnational sample^b^							
Algeria	Abbes, 2016 (2010-2011)	NA	Not engaged in sports	NA	92.8	6-11	293
Egypt	Shady, 2015 (NS)	NA	< 4 hours/week	NA	65.5	9-11	200
Jordan	Haddad, 2009 (NA)	NA	Not very physically nor moderately active	modified Adolescent Wellness Appraisal (AWA)	4.0	12-17	530
KSA	Al-Hazzaa, 2011 (2009-2010)	Arab Teens Lifestyle Study	<1680 METs-min/week	ATLS	61.9	15-19	2908
	Al-Muhaimeed, 2015 (2012)	NA	Not engaging in sports	NA	27.3	6-10	601
	Al-Mutairi, 2015 (2013)	NA	No regular exercise	NA	31.9	15-22	426
	Al-Othman, 2012 (2010)	NA	NA^c^	NA	15.7	6-17	331
	Mahfouz, 2011 (2008)	NA	Less than 30 mins of physical exercise during the previous week	CDC Adolescent Health Survey Questionnaire	34.3	11-19	1869
Kuwait	Shehab, 2005 (NA)	NA	Only performing normal daily routine with some recreational activities or walking slowly and doing no structured exercise	NA	71.3	10-18	400
Lebanon	Nasreddine, 2014 (2009)	NA	Based on weekly frequency: Never^d^	NA	32.6	Mean: 13.06	868
Palestine	Jildeh, 2011 (2002-2003)	The Health Behavior for School-Aged Children Project (HBSC)	<5 days a week	First Palestinian National Health and Nutrition Survey Questionnaire (2000)	77.6	11-16	314
	Arar, 2009 (NA)	NA	No extra-curricular (EC) physical activities	NA	43.3	9-11	180
Sudan	Moukhyer, 2008 (2001)	NA	Not engaging in sports activities	NA	33.4	10-19	1200

^a^GSHS: Global School-based Student Health survey – YRBSS: The Youth Risk Behavior Surveillance System

^b^One study conducted in Lebanon by Shediac-Riskallah (2001) was excluded from the prevalence table as it includes youth (16+ years)

^c^Moderate intensity activities included: playground activities, brisk walking, dancing, and bicycle riding. Higher intensity activities included: ball games, jumping rope, active games involving running and chasing, and swimming

^d^Frequency and type of activities performed along with duration (number of minutes per week)

### Gender differences in physical inactivity

**Fig. 2:**
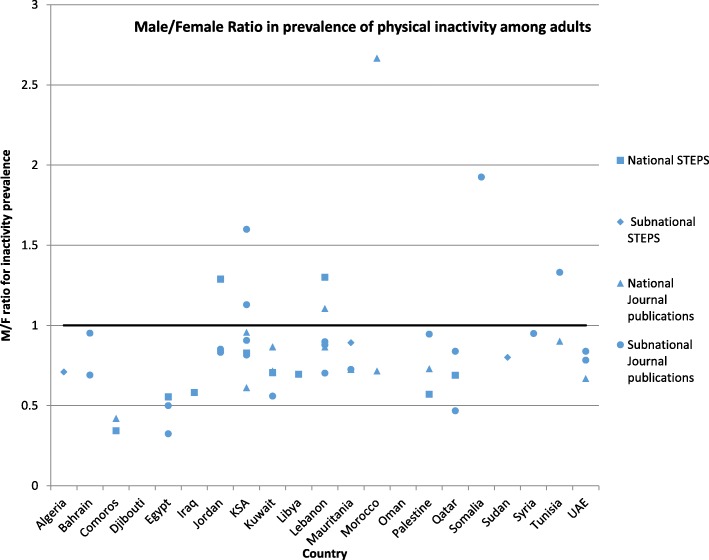
Gender differences in prevalence of physical inactivity among adults

**Fig. 3:**
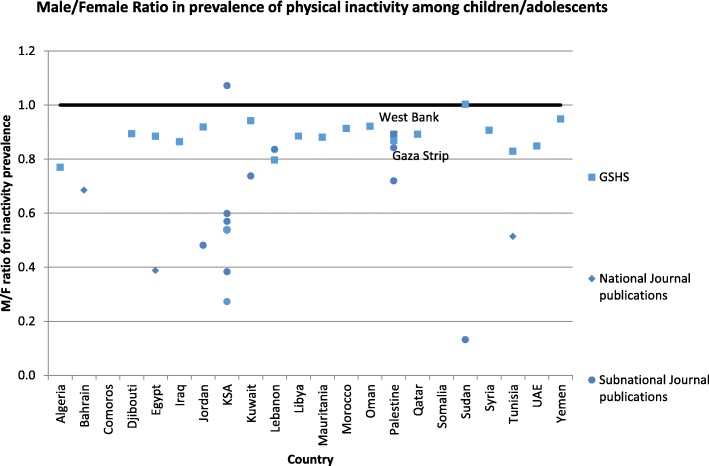
Gender differences in prevalence of physical inactivity among children/adolescents

Where physical activity was reported among men/boys and women/girls, we calculated the M/F ratio of the prevalence of physical inactivity. Figures 2 and 3 show gender ratios among adults and children/adolescents respectively. Overall, the prevalence of inactivity was higher among women/girls in all but 9 studies (8 adults and 1 children/adolescents).

### Socio-demographic and lifestyle determinants

Data from 41 articles about sociodemographic determinants of inactivity were analyzed and results are summarized in Table 6. Inactivity increased with age (18/24 studies), being married (7/10 studies), and urban residence (5/5 studies); it decreased with increased education (14/20 studies) and employment (6/8 studies); parity was positively associated with inactivity in one study. For other sociodemographic determinants, reported associations were inconsistent.

**Table 6:** Factors associated with physical inactivity in Arab countries

	Statistical association with physical inactivity		
	Positive	Negative	Other associations
Age^a^	[13, 29, 35, 36, 38, 39, 69, 73, 83, 92, 100, 135, 143, 153, 176, 218, 221, 231]	[44, 71, 142]	U shape [63, 202] Curvilinear [41]
Marital status	[29, 37, 38, 41, 105, 181, 232]	[42, 79, 221]	
Educational level^a,b^	[35, 54]	[30, 33, 38, 41, 42, 46, 92, 100, 148, 150, 171, 218, 231, 232]	No effect [34, 69, 93]
Employment^a^	[54, 150]	[30, 33, 36, 92, 183, 232]	
SES^a,c^	[12, 35, 46, 75, 105, 232]	[42, 44, 54, 92, 181]	U shape [83]
Urban residence	[35, 36, 43, 77, 150]		
Consuming fruits/vegetables		[33]	
Smoking^a^	[29–32]	[225]	
Alcohol	[30]		
Screen time^d^	[21–28]		
Overweight/ Obesity^e^	[22, 24, 29, 33, 35, 37–39]	[30, 40–42]	No effect [43]
Chronic medical conditions^a^	[29, 34–36]		
Parity	[107]		

^a^The direction of the association between physical inactivity and some variables was not specified in other studies: age [15, 93, 140, 147, 161], educational level [39, 151], employment [29, 37], SES [26, 31, 34, 39, 140], smoking [30], overweight/obesity [15], and chronic medical conditions [28]

^b^Education categorized as iliterate, primary, intermediate, secondary,and university

^c^Socio-economic status (SES): SES score, resources, income, housing type, wealth, Human Development Index, schooling type, domestic help, car ownership

^d^Screen time: Television viewing, computer using, video gaming

^e^The association between underweight and physical inactivity was mentioned in one study and showed a positive association

Several studies found associations between physical inactivity and lifestyle factors. Predictably, screen time was positively associated with physical inactivity in all eight studies that examined this factor [21–28]. Smoking and alcohol were positively associated with physical inactivity [29–32], while consuming fruits and vegetables was negatively correlated [33]. Four studies found a positive association between physical inactivity and chronic medical conditions [29, 34–36].

The studies we reviewed did not report consistent associations between obesity and physical inactivity: 8/13 found a positive association [22, 24, 29, 33, 35, 37–39], four reported the reverse [30, 40–42] and one showed no effect [43].

### Barriers to Physical activity

We examined the subset of studies that investigated barriers to exercise. Some reported reasons were shared with other parts of the world, while others were specific to the Arab region. It is clear that the hot climate of the Arabian Peninsula and Gulf countries limits outdoor physical activity to relatively short seasons and requires special indoor facilities [13, 38, 44–52]. In addition in most countries, the built environment, inadequate public transportation systems, and lack of spaces for walkers or joggers discourage exercise [15, 20, 26, 31, 38, 42, 46, 47, 53–68]. As in other studies, time constraints were mentioned as barriers [15, 26, 28, 30, 31, 40–42, 46, 52, 54, 67, 69–73], in addition to insufficient motivation or interest [25, 26, 34, 44, 46, 54, 71, 73], other priorities [26], and lack of skills [26, 44].

A particularity of the region is the lack of encouragement for physical activity by many parents, who appear to favor educational and spiritual activities over physical activities for their children. Lack of support for physical activity is also noted among friends, peers, and even teachers, in studies conducted in Saudi Arabia, Egypt, and Jordan [15, 20, 24–26, 30, 42, 46, 53–55, 64, 66, 68, 71, 74]. Another regionally specific factor relates to gender constraints: even where fitness facilities are available, as is the case in the more affluent countries of the region, accessibility is a problem, particularly for women.

Lower physical activity among women has been attributed to gender norms, including conservative dress that is not suitable for physical activity, the need for women to be chaperoned in public spaces, and the paucity of gender-segregated fitness facilities [15, 28, 62, 64, 67, 71, 75, 76]. In addition, cultural values put a premium on comfort for both genders, physical exertion is avoided, and public spaces such as streets are not considered appropriate for physical activity. Thus, both general norms and gender norms converge to discourage physical activity [15, 27, 31, 41, 45–47, 54, 60, 62–64, 67, 74, 75, 77–86].

## Discussion

The diversity of definitions and methods among studies published in journals and the fact that only 43/157 studies used validated instruments hampers comparisons of the prevalence and correlates of physical activity, and it is possible that some of the differences we found are artifactual. Using inactivity instead of activity improves the comparability, but it is clear that harmonizing definitions and measurements and considering the multi-dimensional aspects of physical activity would improve the evidence for the region.

Despite these limitations, it is possible to discern some patterns. The results of this review indicate that throughout the region, levels of physical inactivity are very high. Inactivity among adults is 40% or higher in all but five of the fifteen countries with nationally representative surveys; studies with smaller samples suggest even higher levels of inactivity (>60%). Among children and adolescents, inactivity is alarmingly high, around 80% in all national surveys except Tunisia.

High inactivity among children and adolescents is documented in other regions [87, 88] and is a worldwide problem, but the levels of adult inactivity we found in this review compare very unfavorably with those of other regions. Inactivity levels in Europe, the western Pacific, Africa, and southeast Asia are considerably lower (25%, 34%, 28% and 17% respectively [88–90]); they are even lower in South-East Asia and Africa (15% and 21% respectively); the Americas have lower or similar inactivity levels [88–90]. These high levels of inactivity indicate that social circumstances in many countries of the region do not seem to encourage physical activity. Some comparative analyses across countries in the Arab region and outside it have reported that Muslim countries were more likely to be physically inactive, and seemed to suggest that religion constitutes an obstacle to physical activity [91]. This however is not consistent with the diverse interpretations of religious doctrine in the region, and the fact that there are no grounds for arguing that Islamic doctrine is antithetical to exercise. In addition, there is no evidence linking religious observance to lower activity. The one study that compared Muslims and non-Muslims, conducted in Syria, found no significant differences in physical activity between Muslim Syrians and Syrians belonging to other religious groups [92]. Such research highlights the complex interplay among the multiple factors that hinder physical activity.

Physical and social barriers to exercise have been amply documented in multiple Arab countries: the hot weather discourages walking and exertion outdoors; an unfriendly built environment hinders exercise and promotes a car culture; physical exertion is associated with lower status occupations; a premium is placed on comfort; all these contribute to devaluing and discouraging exercise. That parental preferences favor spiritual and educational, over physical activities, and social gatherings are the main leisure activity further contributes to reducing physical activity and encouraging sedentarity [93, 94].

The combination of physical obstacles and low valuations translates into insufficient interest and motivation to exercise, which are documented in multiple countries [20, 46, 52, 68, 90]. A number of studies [24, 40, 71, 74, 95–101] bring out the clustering of health risks within the studied populations, whereby physical activity is one among a set of lifestyle factors that may include energy dense foods, sweet drinks, sedentariness, and unsafe driving. This suggests that lack of knowledge about healthy behaviors in general contributes to inactivity, and emphasizes the role of social activities that are focused on sedentarity and unhealthy snacking. Interestingly, studies [72] that have probed into perceptions of health behaviors have found these to be limited to hygiene, rest and diet, but not physical activity. Thus a combination of material and cultural factors translates into barriers to physical activity at multiple levels and to a lack of awareness and motivation among the population.

A striking result of this analysis is the consistency of gender differences in physical inactivity: in nearly all (45/53) studies conducted among adults and (31/32) among children/adolescents, prevalence of inactivity was higher among women/girls. While traditional religious norms have been reported to potentially define acceptable behaviors for women and preclude exercising, careful qualitative research [76, 102] shows that these are not insurmountable obstacles. Studies show that some women athletes negotiate their involvement in sports even as they continue to wear Islamic clothing, and that decisions to exercise are influenced by new ideas about healthy lifestyles disseminated by professionals. In addition, some studies [103] suggest that ideas about physical activity can become more positive, and that cultural barriers can be overcome when adequate facilities are available.

Studies on ideas about the body report preferences for heavier shapes, especially for men [104] and ethnographic research indicates that there is a normalization of weight gain with increasing age and with maternal status among women [105, 106]. Such notions of the body likely translate into a lack of motivation to exercise and maintain optimal weight across the life cycle for both sexes, and women are vulnerable to weight gain with successive pregnancies. Women's marginalization in segregated societies [107] further pushes them towards a lifestyle centered on hospitality, excessive food consumption and sedentariness. But research also indicates that ideal body shapes can change as a result of exposure to media, as younger women in several countries of the region seem to have adopted thinner body shape preferences [108]. Ideas about exercise can also be transformed by initiatives that provide information about the link between health and exercise, activities that involve women in sports, and efforts to change societal valuations of exertion—and of women [109].

Some initiatives, inspired by those in other regions [110] are underway: policies have been formulated in Oman and Qatar; healthy lifestyles including exercise have been promoted in Morocco, Bahrain and Palestine [111]; some have reported success in improving physical activity in Dubai [89], and Oman [112], while others, such as school-based interventions [113, 114] in Lebanon and Tunisia did not report improvements in physical activity or reductions in screen time. A closer examination of these interventions’ successes and failures can provide useful lessons for future efforts.

## Conclusions

The high levels of inactivity in the region call for considerable efforts to tackle the material and socio-cultural aspects of the cultural context that discourage physical activity. Multi-sectoral efforts are needed, including collaborations among ministries of health, sports, youth and education, as well as wider collaborations that involve sectors such as transport, environment and urban planning [16, 111, 115].

## Additional file

Additional file 1: Medline search strategy. (DOCX 13 kb)

## Abbreviations

ATLS: Arab Teens Lifestyle Study
DALYs: Disability Adjusted Life Years
GPAQ: Global Physical Activity Questionnaire
GSHS: Global School-based Student Health Surveys
IPAQ: International Physical Activity Questionnaire
KPAS: Kaiser Physical Activity Survey
KSA: Kingdom of Saudi Arabia
LASA: Longitudinal Aging Study Amsterdam
MESA: Multi-Ethnic Study of Atherosclerosis questionnaire
ODK: Open Data Kit
PACE+: Patient-Centered Assessment and Counseling for Exercise Plus Nutrition
PRISMA: Preferred Reporting Items for Systematic Reviews and Meta-Analyses
SSCI: Social Sciences Citation Index
UAE: United Arab Emirates
WHO STEPS: World Health Organization STEPwise approach to Surveillance
WHO: World Health Organization
WHS: World Health Surveys
YRBSS: The Youth Risk Behavior Surveillance System
