# Supplementary material for: Physical inactivity, gender and culture in Arab countries: a systematic assessment of the literature
Source: BMC Public Health. 2018 May 18;18:639. doi: 10.1186/s12889-018-5472-z (PMC5960209; doi:10.1186/s12889-018-5472-z)
Supplement: Supplementary file 1 — Medline search strategy. (DOCX 13 kb) [file 12889_2018_5472_MOESM1_ESM.docx]

**Additional File** 1

Format: Word document .doc

Title: Medline search strategy

1. Physical inactivity.ti,ab or sedentary lifestyle/ or sedentary lifestyle.ti,ab or or sedentary behavior?r.ti,ab or physical activit*.ti,ab or exercise/ or exercise.ti,ab or sports/ or sports.ti,ab or physical fitness/ or physical fitness.ti,ab or vigorous activity.ti,ab or physical exertion/ or physical exert*.ti,ab or ((physical* or sport* or exercise* or game*) adj3 (activit* or exercise* or exert* or fit or fitness or game* or endurance* or inactiv* or train* or training)).ti,ab
2. Arab world/ or Arabs.mp. or Algeria/ or Algeria*.mp. or Bahrain/ or Bahrain*.mp. or Comoros/ or Comoros.mp. or Comorian*.mp. or Djibouti/ or Djibouti*.mp. or Egypt/ or Egypt*.mp. or Iraq/ or Iraq*.mp. or Jordan/ or Jordan*.mp. or Kuwait/ or Kuwait*.mp. or Lebanon/ or Lebanese.mp. or Libya/ or Libya*.mp. or Mauritania/ or Mauritania*.mp. or Morocco/ or Morocc*.mp. or Oman/ or Oman*.mp. or Palestine/ or Palestin*.mp. or occupied Palestinian territor*.mp. or West Bank.mp. or Gaza.mp. or Qatar/ or Qatar*.mp. or Saudi Arabia/ or Saudi*.mp. or Somalia/ or Somali*.mp. or Sudan/ or Sudan*.mp. or Syria/ or Syria*.mp. or Tunisia/ or Tunisia*.mp. or United Arab Emirates.mp. or Emirat*.mp. or Yemen/ or Yemen*.mp. or gulf cooperation council.mp. or gulf countr*.mp. or middle east.mp. or Middle East/ or Africa, Northern/ or North Africa*.mp. or (Middle East and North Africa).mp.
3. epidemiology/ or epidemiolog*.mp or prevalence/ or prevalen* or incidence/ or inciden*.mp or Vital Statistics or vital statistics.mp
4. Burden of disease.mp or disease burden.mp or morbidity/ or morbidit*.mp or mortality/ or "cause of death"/ or mortality, premature/ or survival rate/or Quality-Adjusted Life Years/ or quality-adjusted life years.mp or QALY.mp or disability-adjusted life years.mp or DALY.mp or YLD.mp or years of life lost.mp or YLL.mp or years lived with disability.mp or years lived with disease.mp or cost of illness/
5. (social or socio* or cultur*).mp.
6. 3 or 4 or 5
7. 1 and 2 and 6
